# Molecular Order Induced Charge Transfer in a C_60_-Topological Insulator Moiré Heterostructure

**DOI:** 10.1021/acs.nanolett.4c06294

**Published:** 2025-01-13

**Authors:** Ram Prakash Pandeya, Konstantin P. Shchukin, Yannic Falke, Gregor Mussler, Abdur Rehman Jalil, Nicolae Atodiresei, Eddwi H. Hasdeo, Alexander Fedorov, Boris V. Senkovskiy, Daniel Jansen, Giovanni Di Santo, Luca Petaccia, Alexander Grüneis

**Affiliations:** † Institut für Festkörperelektronik, Technische Universität Wien, Gußhausstraße 25, 1040 Vienna, Austria; ‡ II. Physikalisches Institut, Universität zu Köln, Zülpicher Strasse 77, 50937 Köln, Germany; ¶ Peter Grünberg Institut (PGI-9), Forschungszentrum Jülich, D-52425 Jülich, Germany; § Peter Grünberg Institut (PGI-1), Forschungszentrum Jülich, D-52425 Jülich, Germany; ∥ Department of Physics and Materials Science, Universite’ du Luxembourg, L-1511 Luxembourg, Luxembourg; ⊥ Research Center for Quantum Physics, National Research and Innovation Agency, 15314 South Tangerang, Indonesia; # Leibniz Institute for Solid State and Materials Research, Helmholtzstraße 20, 01069 Dresden, Germany; @ Elettra Sincrotrone Trieste, Strada Statale 14 km 163.5, 34149 Trieste, Italy

**Keywords:** topological insulators, fullerene, angle-resolved photoemission, Raman, photoluminescence

## Abstract

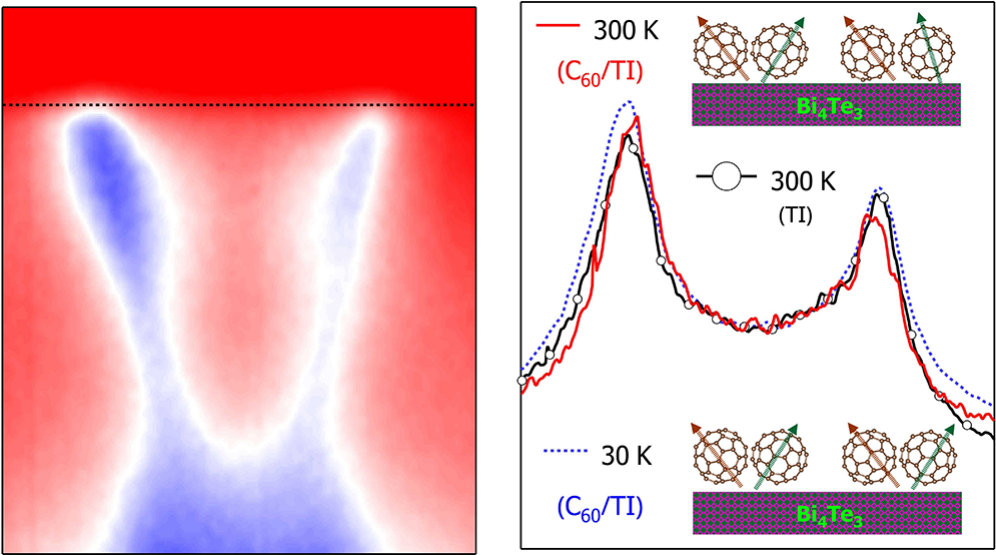

We synthesized and spectroscopically investigated monolayer (ML) C_60_ on the topological insulator (TI) Bi_4_Te_3_. This C_60_/Bi_4_Te_3_ heterostructure is characterized by an excellent translational order in a novel (4 × 4) C_60_ superstructure on a (9 × 9) cell of Bi_4_Te_3_. Angle-resolved photoemission spectroscopy (ARPES) of C_60_/Bi_4_Te_3_ reveals that ML C_60_ accepts electrons from the TI at room temperature, but no charge transfer occurs at low temperatures. This temperature-dependent doping is further investigated by Raman spectroscopy, photoluminescence (PL), and calculations of C_60_/Bi_4_Te_3_. At low temperatures, Raman spectroscopy and PL show a dramatic intensity increase of the C_60_-related signal, suggesting a transition to a rotationally ordered state. Calculations explain the charge transfer by C_60_ adsorption to Bi_4_Te_3_ surface defects. The temperature dependence of the charge transfer is attributed to the orientational order of C_60_. The electron affinity of C_60_ increases at low temperatures due to the freezing of the rotational motion.

Organic thin films on metals or semiconductors form interfaces with exciting optoelectronic properties that are controlled by charge transfer and molecular order.^1^ Two crucial quantities for obtaining defined interfaces are the relative strengths of the molecule–molecule versus the molecule–substrate interactions.^2^ Molecule–substrate interaction imprints molecular order but is detrimental to inheriting the molecular properties from the interface electronic states. The surfaces of bulk metals and semiconductors often strongly bond to molecules, because dangling bonds are present on their surfaces. Their structure is complicated by surface reconstructions, change in molecular shape, and geometrical frustration.^3−5^ Van-der-Waals substrates have a relatively weaker interaction with deposited molecules; for example, for pentacene on graphene, a coverage and substrate doping dependent molecular orientation has been observed.^6^ For C_60_/graphene, p-doping^7,8^ and a Fermi-level-dependent charge transfer to graphene were observed.^9^ Van-der-Waals epitaxy and interaction of organic molecules have been investigated for PTCDA on graphene,^10^ C_60_ on hBN,^11,12^ C_60_ on WSe_2_,^13^ borophene on hBN,^14^ and PTCDA on borophene.^15^ Such heterostructures are useful for transistors and optoelectronic devices.^16−21^ The organic layer/topological insulator (TI) system provides a new material system that allows us to study the interaction of the molecules with topologically protected surface electronic states. Molecule–TI interactions have been investigated for monolayers of MnPc,^22^ CuPc,^22,23^ and CoTBrPP^24^ on Bi_2_Te_3_ and CoPc,^24,25^ H_2_Pc,^26^ and PTCDA^27^ on Bi_2_Se_3_. The electronic structure of highly ordered C_60_ with a thickness of 50 Å on Bi_2_Se_3_ TI has been investigated, and good agreement with density functional theory (DFT) calculations was found.^28^ The large C_60_ thickness prevented the study of the structure of the buried interface. Although single-layer C_60_ on Bi_2_Se_3_ has been studied, revealing no significant change in substrate band structure, the effect of long-range molecular ordering is yet to be explored.^26^ Therefore, the charge transfer between the TI and C_60_, the effect of C_60_ on the dispersion of the TI surface state, the molecular orientation, and the optical properties of the interface are not known. Moreover, the role of defects of the TI surface in the charge transfer to the organic adsorbate layer has not been investigated.^29,30^ Thick films of C_60_ have a structural phase transition from face centered cubic (fcc) to simple cubic (sc) at 250 K.^31,32^ For fcc (sc), neighboring C_60_ have different (identical) orientation.^32,33^ This phase transition affects the Raman and photoluminescence (PL) spectra. Below the transition temperature the Ag(2) (pentagonal pinch) Raman mode hardens and becomes more intense and sharper^31,34^ and the PL intensity increases.^35^ It is unclear whether that phase transition occurs for a C_60_ monolayer and how it is affected by the substrate.

The low-energy electron diffraction (LEED) pattern of MBE-grown^36^ Bi_4_Te_3_ is shown in Figure 1a along with sketches of the LEED pattern, the (0001) surface of Bi_4_Te_3_, and the first Brillouin zone (BZ). Figure 1b shows the hexagonally warped, n-type Fermi surface of Bi_4_Te_3_ measured by angle-resolved photoemission spectroscopy (ARPES) along with a tight-binding (TB) fit to ARPES maxima.^37,38^ From the TB fit, we determine the value of the ratio between hexagonal warping λ to Fermi velocity *v* of λ/*v* = 27 Å^2^. Using the Fermi surface size, we estimate the carrier concentration as *n* = 8.98 × 10^12^ cm^–2^. Figure 1c depicts an ARPES scan through the BZ center along *ΓK* in the vicinity of *E*_*F*_. The size and shape of the Fermi surface are qualitatively similar to bulk Bi_4_Te_3_.^38^

**Figure 1:**
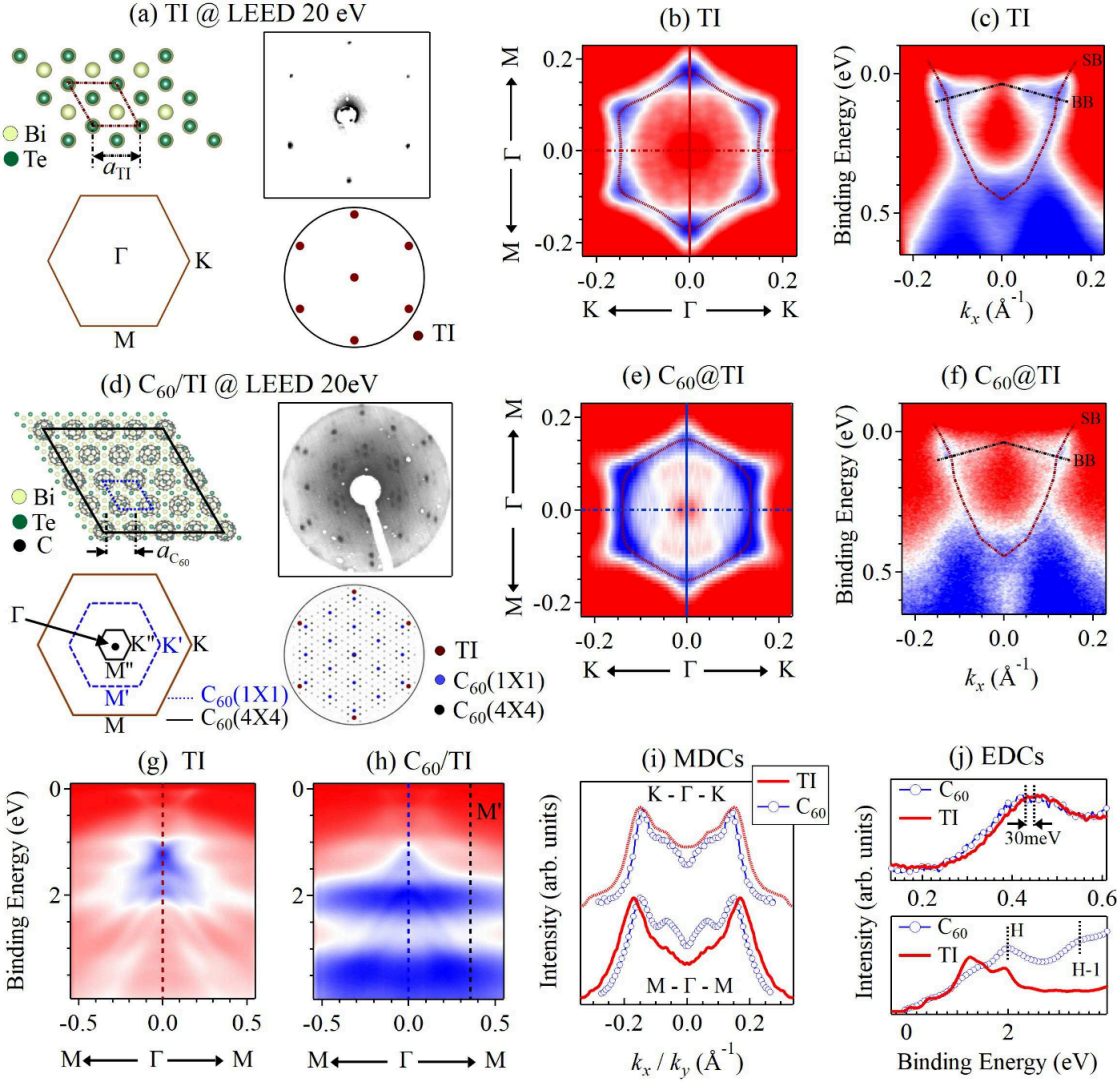
(a) Upper row: surface crystal structure and LEED pattern of Bi_4_Te_3_. Lower row: Bi_4_Te_3_ BZ and a sketch of the LEED pattern. (b) Fermi surface of Bi_4_Te_3_ and tight-binding (TB) fit of the TI surface state (red). (c) ARPES of Bi_4_Te_3_ along the *ΓK* direction. The surface bands (SB) and bulk bands (BB) are indicated. (d) Upper row: surface unit cell with the 4 × 4 C_60_ superstructure on 9 × 9 TI, the BZ, and a sketch of the surface unit cell. Lower row: LEED pattern of monolayer C_60_ on Bi_4_Te_3_ and sketch of the LEED pattern reflexes from Bi_4_Te_3_, C_60_, and C_60_ superstructure. (e) Fermi surface of C_60_/Bi_4_Te_3_ along with a TB fit of the TI surface state (red). (f) ARPES scan of C_60_/Bi_4_Te_3_ along the *ΓK*. (g and h) ARPES of Bi_4_Te_3_ and C_60_/Bi_4_Te_3_ in a wide energy range including the HOMO and HOMO–1 of C_60_ at 2 and 3.5 eV, respectively. (i) Momentum dispersion curves (MDCs) at *E*_*F*_ of Bi_4_Te_3_ and C_60_/Bi_4_Te_3_ along *ΓK* and *ΓM*. (j) Energy distribution curves (EDCs) of Bi_4_Te_3_ and C_60_/Bi_4_Te_3_ at Γ in a narrow energy range around the Dirac point and in a wide energy range. The labels H and H–1 indicate the HOMO and HOMO–1 levels. All ARPES data are taken at 300 K with *hν* = 21.2 eV.

Figure 1d depicts the LEED pattern of one ML of C_60_ evaporated on Bi_4_Te_3_ along with sketches of the LEED pattern and interface as well as the reciprocal lattice of the new supercell. LEED shows the diffraction spots of Bi_4_Te_3_ and the additional spots that come from C_60_. From LEED we observe that the reciprocal lattice vector corresponding to the C_60_ lattice is almost half of a Bi_4_Te_3_ reciprocal lattice vector. Since the lattice constant of the C_60_ layer is not close to an integer multiple of the lattice constant of Bi_4_Te_3_, a large moiré pattern forms. The set of diffraction spots of weak intensity around each C_60_ diffraction spot are due to that moiré pattern that C_60_ forms on the Bi_4_Te_3_ surface. In the lower right panel of Figure 1d, the diffraction spots are color-coded according to their origin. The pattern inherits the same orientation as the interface and is due to the arrangement of C_60_ in a (9 × 9) superstructure with respect to the TI lattice, as sketched in the upper left panel of Figure 1d. The new moiré supercell contains 16 C_60_ molecules and is defined by a lattice vector of ∼4 nm in length. The bottom left panel of Figure 1d depicts the BZ of Bi_4_Te_3_ along with the C_60_ and the moiré BZs. Based on *a*_Bi_4_Te_3__, the lattice constant of Bi_4_Te_3_, we estimate the C_60_ lattice on Bi_4_Te_3_ to be (9/4) × *a*_Bi_4_Te_3__ = 10.15 Å. The C_60_ lattice constant in the fcc structure is *a*_C_60__^*fcc*^ = 14.12 Å.^39^ Hence, the lattice constant of a hexagonal lattice defined on the fcc(111) plane is *a*_C_60__^*fcc*^/√2 = 10.01 Å, implying that the C_60_ monolayer on Bi_4_Te_3_ is strained by 1.4%.

The Fermi surface of C_60_/Bi_4_Te_3_ measured by ARPES is shown in Figure 1e. Performing a TB fit, λ/*v* = 22 Å^2^ and *n* = 7.59 × 10^12^ cm^–2^ are obtained. Compared to pristine Bi_4_Te_3_, Bi_4_Te_3_ with C_60_ on its surface has smaller hexagonal warping and is p-doped. We estimate an average charge transfer of 0.035 hole per C_60_. Figure 1f shows the ARPES scan of C_60_/Bi_4_Te_3_. The ARPES scans of Figures 1g and 1h show the energy band structures in a wide energy range of pristine Bi_4_Te_3_ and C_60_/Bi_4_Te_3_, respectively. The highest occupied molecular orbital (HOMO) and HOMO–1 energy levels from C_60_ appear at binding energies of ∼2 eV and ∼3.2 eV, respectively. Their 2D energy dispersions are also measured by ARPES with linearly polarized light (Supporting Information). In Figure 1i, the momentum distribution curves (MDCs) at the Fermi energy in *ΓK* and *ΓM* directions are shown for Bi_4_Te_3_ and C_60_/Bi_4_Te_3_. The Fermi wavevector reduces for C_60_/Bi_4_Te_3_ in both directions. This is consistent with the observed reduction of the Fermi surface after C_60_ evaporation. In Figure 1j, we show the energy distribution curves (EDCs) of Bi_4_Te_3_ and C_60_/Bi_4_Te_3_ at the Γ-point and across the HOMO and HOMO–1 levels. The direct comparison of EDCs for Bi_4_Te_3_ and C_60_/Bi_4_Te_3_ shows a shift of the EDC maximum toward the Fermi level as a result of C_60_ deposition.

Figure 2a depicts Raman spectra recorded at 300 and 80 K for one (0.5) ML of C_60_/Bi_4_Te_3_ along with the Raman spectra taken on pristine Bi_4_Te_3_. The Raman intensity of Bi_4_Te_3_ is negligible compared to the C_60_ Raman intensity. At 300 K, a broad Ag(2) C_60_ Raman mode in the spectrum of C_60_/Bi_4_Te_3_ can be detected at around 1450 cm^–1^. At 80 K, the Ag(2) mode shifts to higher energy and becomes narrower and more intense. The energy upshift, increased Raman intensity, and narrower peak at low temperatures are similar to the fcc to sc transition in μm thick C_60_ films.^31,32,34^ At temperatures below the phase transition (250 K), the rotational motion of C_60_ is frozen. In a previous work^34^ using a 514 nm laser, the measured Raman intensity of the Ag(2) mode increases by a factor ∼2 when cooled from 280 K to 40 K. Let us now look at the intensity ratio of the 1450 cm^–1^ Raman peaks at 80 K and at 300 K in Figure 2a. Comparing the peak Raman intensity ratios for the 0.5 (1.0) ML curves for 80 and 300 K, we obtain 5.9 (5.5) (±0.5). We believe that the differences of the temperature-dependent Raman intensity increase of C_60_/Bi_4_Te_3_ and thick C_60_ films can be explained by the dielectric environments, band gaps, resonance conditions, and de-excitation processes.

**Figure 2:**
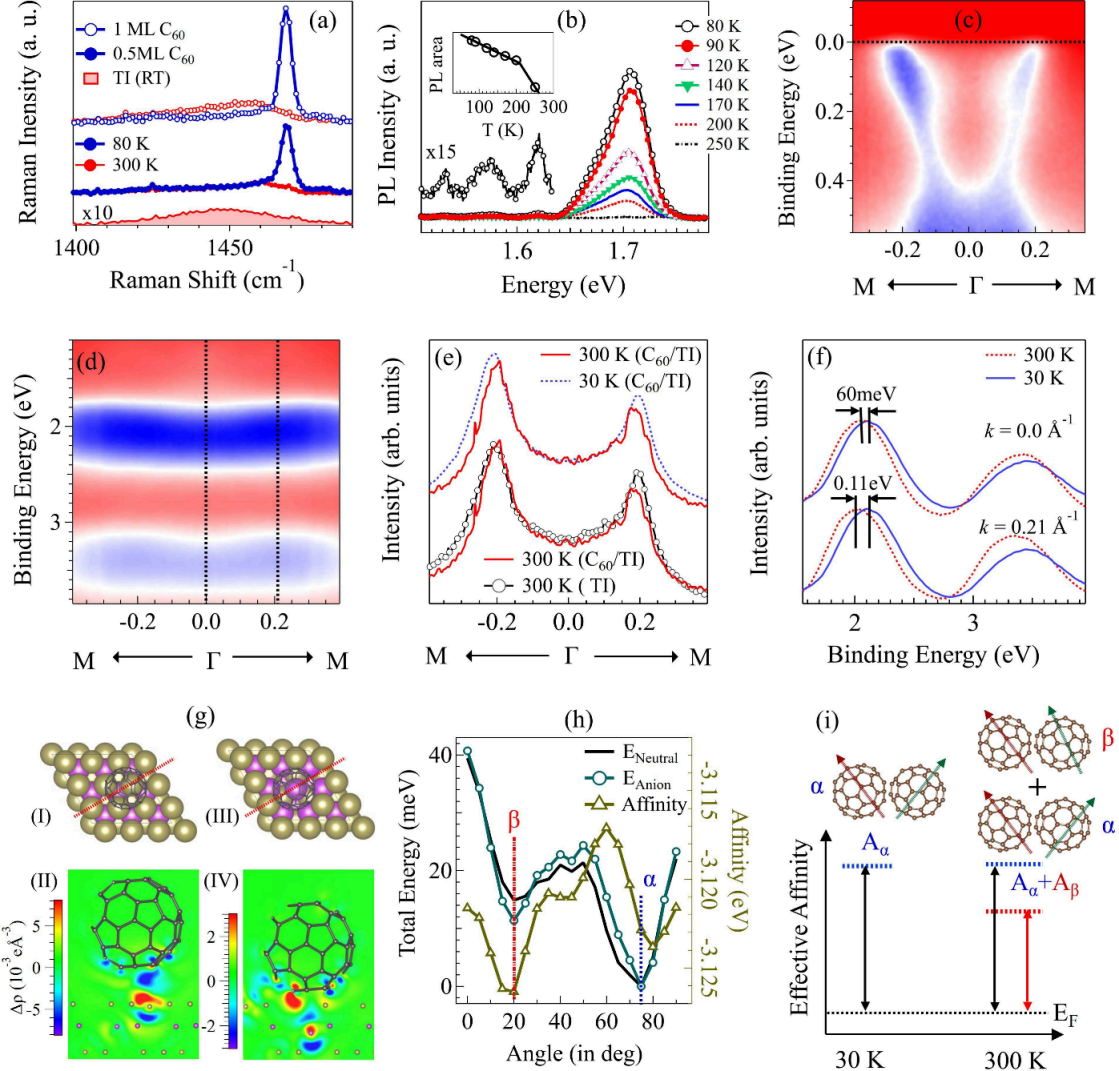
(a) Raman spectra (λ_*exc*_ = 532 nm) of C_60_/Bi_4_Te_3_ at 80 K and at 300 K for 0.5 and 1 ML C_60_ coverages (the C_60_ Ag(2) mode is shown) and the Bi_4_Te_3_ Raman spectrum for comparison. (b) Temperature-dependent photoluminescence (PL) spectra of C_60_/Bi_4_Te_3_ (λ_*exc*_ = 532 nm). The inset shows the integrated PL intensity. The magnified region shows the phonon side peaks. (c, d) ARPES of C_60_/Bi_4_Te_3_ at 30 K in the energy regions of the TI surface state and the HOMO/HOMO–1 states. (e) MDCs at *E*_*F*_ of Bi_4_Te_3_ at 300 K and C_60_/Bi_4_Te_3_ at 300 and 30 K taken along ΓM. (f) EDCs of C_60_/Bi_4_Te_3_ through HOMO and HOMO–1 bands at two wavevectors measured at 30 and 300 K. (g) Adsorbed C_60_ onto (I) Te-terminated Bi_4_Te_3_ and (II) the adsorption induced charge accumulation difference *Δρ* (red and blue are positive and negative *Δρ*, respectively) along the red line in (I). (III) and (IV) depict the geometry and *Δρ* for C_60_ on a Te triple vacancy. (h) Total energy of a C_60_ dimer (neutral and anion) (left) and electron affinity (right) vs the rotation angle between the two constituent C_60_. Angles α and β denote the global and local minima of the total energy. (i) Schematic diagram of dimers with angles α and β between C_60_. At low temperature, all dimers are in the ground state with the relative rotational angle α. At high temperatures, the state with angle β between C_60_ becomes populated. State β has a lower *E*_*A*_, explaining why C_60_ is ionized more easily at higher temperature.

Figure 2b depicts PL spectra at temperatures between 300 and 80 K. Upon cooling below ∼250 K, the PL intensity at 1.7 eV increases. Matus et al.^35^ relate the PL increase of C_60_ films at low temperature to the phase transition from fcc to sc. At temperatures above the phase transition, the rotation of C_60_ causes electron–hole excitation to remain on one C_60_. Adjacent C_60_ molecules rotate, and there is insufficient overlap between them. Thus, the excitation cannot be transferred and is zero-dimensional. If the temperature is decreased, the fullerenes do not rotate and the overlap between neighboring C_60_ increases. That is, the electronic structure becomes three-dimensional, and an exciton delocalizes over more than one C_60_. Excitons can diffuse through the crystal until they recombine radiatively at a defect.^35^ Additionally, the PL increase at low temperatures could be related to the loss of nonradiative decay channels that require temperature activation and the absence of electron transfer from the Bi_4_Te_3_ to C_60_. The PL spectra of C_60_ have side peaks due to coupling to phonons that are red-shifted from the main peak by the phonon energy. We find peaks shifted by 80, 130, and 240 meV from the main PL peak (magnified spectrum in Figure 2b). We compare the peak energies to the phonons of C_60_ and find a reasonable agreement with calculated phonons^40^ at 78 meV (*H*_*u*_^5^), 127 meV (*T*_3*u*_^3^), and the third overtone of *H*_*u*_^5^ at 234 meV. Note that vibronic and phonon sidebands have also been observed previously in 1 μm thick C_60_ films.^41^

Figures 2c and 2d depict ARPES of C_60_/Bi_4_Te_3_ recorded at 30 K in energy ranges of the Dirac cone of Bi_4_Te_3_ and the molecular C_60_ bands, respectively. Figure 2e shows a comparative MDC analysis of the ARPES spectra of pure Bi_4_Te_3_ and C_60_/Bi_4_Te_3_ at 300 K and C_60_/Bi_4_Te_3_ at 30 K carried out on the same sample. MDCs at the Fermi level along the ΓM direction are shown. This Bi_4_Te_3_ surface was prepared on a different Bi_4_Te_3_ film than the surface shown in Figure 1, resulting in a slightly different carrier concentration. The analysis in Figure 2e affirms the previous conclusion from Figure 1i; that is, upon C_60_ deposition, the Fermi surface size of Bi_4_Te_3_ shrinks. From Figure 2e we also observe that, upon cooling C_60_/Bi_4_Te_3_ to 30 K, the Fermi surface size increases almost back to the original value of pristine Bi_4_Te_3_; that is, we have a temperature-dependent charge transfer between C_60_ and Bi_4_Te_3_. Figure 2f depicts the EDCs of the ARPES data from Figure 2c at 300 K and at 30 K. The two intense peaks correspond to the HOMO and the HOMO–1 of C_60_. It can be seen that the HOMO and HOMO–1 bands shift to higher binding energy by 60 meV and by 110 meV, respectively, when cooling down the sample from 300 K to 30 K. This downshift is not due to electron doping of C_60_ because also the LUMO (not visible in ARPES) shifts to a higher energy when the sample is cooled. The LUMO upshift follows from DFT calculations. As a consequence of the concurrent downshift of HOMO and upshift of LUMO, C_60_ is p-doped. In order to rationalize the observed findings regarding the charge transfer, we consider two effects. First, we calculate the charge transfer of C_60_ to the unfaulty TI surface and to a realistic TI surface with Te vacancies. Second, we calculate the temperature-dependent change transfer between C_60_ and Bi_4_Te_3_ by DFT using a C_60_ dimer model with a temperature-dependent rotational angle between the two C_60_. Figure 2g (subfigures I and II) shows DFT calculations of the geometry and the electron density of an isolated C_60_ adsorbed onto the Te-terminated Bi_4_Te_3_ surface. C_60_ adsorbs 3.09 Å above the surface with respect to a Te surface atom. The interaction energy of the physisorbed C_60_ onto the TI surface is −0.716 eV. From a Bader charge density analysis of C_60_/Bi_4_Te_3_, we identify an electron transfer from C_60_ to Bi_4_Te_3_ of 0.042 electron, suggesting that the Bi_4_Te_3_ substrate is slightly n-doped, in contradiction to the ARPES results. In Figure 2g (subfigures III and IV), we consider C_60_ adsorbed to a triple Te vacancy defect on Bi_4_Te_3_. In this geometry, C_60_ is 1.10 Å above the surface and can directly interact with Bi. As a consequence, the interaction energy of C_60_ on a triple vacancy equals −1.229 eV. Performing a Bader charge density analysis we find that 0.208 electron is transferred from Bi_4_Te_3_ to one C_60_, explaining ARPES results at room temperature. Note that for an antisite defect where a Te atom is replaced by a Bi atom^29,30^ in the topmost layer, we would also expect electron transfer from Bi_4_Te_3_ to C_60_ and thus consistency with ARPES observations.

We now consider the effect of the rotational order of adjacent C_60_ on the charge transfer. The electron affinity *E*_*A*_ is defined as the difference in the total energy between the negatively charged system and the neutral system. For the case of strong electron acceptors such as C_60_, the negatively charged system (with one extra electron in the LUMO) has a lower total energy, which leads to negative values of *E*_*A*_. The calculations of the realistic C_60_/Bi_4_Te_3_ supercell are prohibitively demanding, and we thus consider a C_60_–C_60_ dimer as a model to qualitatively show the effect of relative rotation. Figure 2h depicts the total energy and *E*_*A*_ of the C_60_ dimer as a function of the C_60_ relative rotation angle. There is one global minimum at a relative rotational angle of ∼75° (α-phase). Another local minimum is located at a relative rotational angle of ∼20° (β-phase). We therefore expect that at low temperatures all C_60_ are in the α-phase, and at room temperature, some of the C_60_ molecules occupy the β-phase due to small energy difference between the two phases. We consider the electron affinity *E*_*A*_ of these two phases in order to explain the observed temperature-dependent charge transfer to Bi_4_Te_3_ from C_60_. For the α-phase, the *E*_*A*_ = −3.123 eV and for the β-phase, *E*_*A*_ = −3.126 eV. The smaller value of *E*_*A*_ for the β-phase means that in this phase the energy gain per C_60_ is larger, if an electronic charge is transferred from Bi_4_Te_3_. That is, if more C_60_ dimers are in the β-phase, then they can accept more electrons. These calculations qualitatively explain the experimental observation that at 300 K we have a smaller Bi_4_Te_3_ Fermi surface than at 30 K. The limitations of the calculations are the simplification of using the C_60_ dimer versus the real interface, which ignores the effects of each C_60_ having six nearest C_60_ neighbors and the effect of substrate interaction. Figure 2i depicts *E*_*A*_ for the α- and β-phases of the C_60_ dimer as a function of temperature. At 30 K the α-phase contributes and C_60_ dimers in this phase have a high *E*_*A*_. As a consequence, it is energetically not favorable to transfer an electron from Bi_4_Te_3_ to C_60_. At 300 K, also the β-phase contributes, which lowers the effective *E*_*A*_, making it energetically favorable to transfer electrons from Bi_4_Te_3_ to C_60_.

In conclusion, the moiré structure at the C_60_/Bi_4_Te_3_ interface could be observed in ML C_60_ films on Bi_4_Te_3_ by LEED. The Raman modes and the PL response of C_60_ in the C_60_/Bi_4_Te_3_ heterostructure are enhanced at 30 K due to freezing of C_60_ rotation. ARPES of C_60_/Bi_4_Te_3_ at 300 K reveals an electron transfer from Bi_4_Te_3_ to C_60_ but no electron transfer at 30 K. Calculations of realistic C_60_ adsorption geometries on triple Te vacancies of Bi_4_Te_3_ and the temperature-dependent *E*_*A*_ of a C_60_ dimer explain the experimental results. Our work illustrates the complex temperature-dependent interplay between charge transfer and molecular order and is relevant for describing molecule–surface interactions. Future experiments in this direction could use magnetic fullerenes on TI or study the interface between TI and superconducting alkali metal doped fullerene film (e.g., TI/Rb_3_C_60_).

## Supporting Information Available

ARPES characterization of C_60_/Bi_4_Te_3_ using polarized synchrotron radiation; experimental and theoretical methods; calculated interaction energies and charge transfer between C_60_ and the defective Bi_4_Te_3_ surface; analysis of hole doping of Bi_4_Te_3_ and the charge transfer per C_60_; evaluation of Te triple vacancy density; dependence of charge transfer on the C_60_–Bi_4_Te_3_ distance (PDF)
