# Supplementary material for: Molecular Order Induced Charge Transfer in a C60-Topological Insulator Moiré Heterostructure
Source: Nano Lett. 2025 Jan 13;25(3):1220–5. doi: 10.1021/acs.nanolett.4c06294 (PMC11760159; doi:10.1021/acs.nanolett.4c06294)
Supplement: Supplementary file 1 — nl4c06294_si_001.pdf [file nl4c06294_si_001.pdf]

Supporting information for Molecular order induced charge transfer in a C_{60}-topological insulator moiré heterostructure

Ram Prakash Pandeya,[*,†,‡] Konstantin P. Shchukin,[†,‡] Yannic Falke,[‡] Gregor Mussler,[¶] Abdur Rehman Jalil,[¶] Nicolae Atodiresei,[§] Eddwi H. Hasdeo,[‖,⊥] Alexander Fedorov,[‡,#] Boris V. Senkovskiy,[‡] Daniel Jansen,[‡] Giovanni Di Santo,[@] Luca Petaccia,[@] and Alexander Grüneis[*,†,‡]

†*Institut für Festkörperelektronik, Technische Universität Wien, Gußhausstraße 25, 1040 Vienna, Austria*

‡*II. Physikalisches Institut, Universität zu Köln, Zülpicher Strasse 77, 50937 Köln, Germany*

¶*Peter Grünberg Institut (PGI-9), Forschungszentrum Jülich, D-52425 Jülich, Germany*

§*Peter Grünberg Institut (PGI-1), Forschungszentrum Jülich, D-52425 Jülich, Germany*

‖*Department of Physics and Materials Science, Universite' du Luxembourg, L-1511 Luxembourg, Luxembourg*

⊥*Research Center for Quantum Physics, National Research and Innovation Agency, 15314 South Tangerang, Indonesia*

\# *Leibniz Institute for Solid State and Materials Research, Helmholtzstraße 20, 01069 Dresden, Germany*

@*Elettra Sincrotrone Trieste, Strada Statale 14 km 163.5, 34149 Trieste, Italy*

E-mail: ram.pandeya@tuwien.ac.at; alexander.grueneis@tuwien.ac.at

Abstract

Further ARPES characterization of the two-dimensional energy band structure of the C_{60} derived bands is provided. The experimental and theoretical methods used are explained. The interaction energies and the charge transfer between C_{60} and the defective Bi_4Te_3 surface are calculated. Experimental results of hole doping of Bi_4Te_3 are analyzed and the charge transfer per C_{60} is estimated. The Te triple vacancy density is evaluated. The dependence of the charge transfer on C_{60}-Bi_4Te_3 distance is calculated.

C_{60} derived electron energy band structure of C_{60}/Bi_4Te_3

Here we show a detailed study of the molecular energy band dispersion of C_{60} derived bands with HOMO and HOMO-1 character of the C_{60}/Bi_4Te_3 heterostructure. In the isolated C_{60} molecule, these orbitals are ten-fold degenerate but in the solid this degeneracy is lifted.[1] In order to disentangle the sub-band structure, we perform ARPES using linearly $s-$ and $p-$polarized synchrotron radiation. The polarized light couples preferentially to one of the several sub-bands and allows us to probe the sub-bands of HOMO and HOMO-1 levels individually. Figures S1a,b and S1c,d show the ARPES spectrum of C_{60}/Bi_4Te_3 measured in the vicinity of the HOMO and HOMO-1 energy levels employing $p(s)$-polarized light along ΓM/ΓK-directions, respectively. Both the HOMO and HOMO-1 bands reveals a significant band-dispersion due to long-range crystalline ordering. A clear electron- and hole-like parabolic dispersion relation can be observed with the p- and s-polarized lights, respectively. This reveals presence of at least two oppositely dispersive bands at both HOMO and HOMO-1 energy levels. The measured dispersion band width of the HOMO and HOMO-1 bands with p-polarized light source are $\sim$ 0.2 and 0.1 eV, respectively. Interestingly, the hole-like band observed with the s-polarized light source are weakly dispersive. It is clear that bands having dominant orbital character defined parallel to the interface plane are more dispersive than the out-of-plane direction. This is expected from the band-narrowing effect along the

out-of-plane direction due to absence of more than one C_{60} molecules towards vacuum.

Figure S1i-l shows the 2D ARPES constant energy contours measured in the vicinity of HOMO and HOMO-1 energy levels using linearly polarised photons. In addition to the dispersion of the C_{60} bands, the 2D ARPES constant energy maps reveals an angular anisotropy for both HOMO and HOMO-1 bands. The hexagonal symmetry of constant energy ARPES maps measured using p polarisation confirms the angular anisotropy of both C_{60} HOMO and HOMO-1 dispersive bands. Despite the bands measured using s-polarised light are weakly dispersive, the hexagonal anisotropy of the constant energy ARPES contours could be seen in Figure S1k-l. Clearly, the ten-fold degeneracy of the C_{60} is splitted into at least two energy levels due to highly ordered hexagonal crystalline structure on Bi_4Te_3. In addition, the equi-energy contours also show hexagonally anisotropic dispersive bands originating from the molecular orbitals.

Experimental methods

Bi_4Te_3 condenses in a rhombohedral crystal structure (R$\bar{3}$m space group) with the unit cell consisting of stacked quintuple layers (QL) with Bi_2Te_3 stoichiometry and Bi-Bi bilayers (BL). The 20 nm Bi_4Te_3 thin films used in this study are grown in (0001) direction on Si(111) in ultra-high vacuum (UHV) with a base pressure $< 5 \times 10^{-10}$ mbar.[2] The (0001) surface of Bi_4Te_3 has a hexagonal lattice with lattice constant $a_{\mathrm{hex}} = 4.501$ Å as determined by X-Ray Diffraction (XRD) experiments.[3] Before exposure to air, the films were capped with an amorphous 2 nm Al_2O_3 layer.

In a separate preparation chamber of the ARPES system with a base pressure $< 5 \times 10^{-10}$ mbar, the capping layer was removed by means of sputtering and annealing cycles with temperatures as high as 650 K until no improvement of the LEED spectrum was distinguishable any more. The sharp LEED reflexes indicate a clean surface and high crystallinity over the whole $1 \times 1\,\mathrm{cm}^2$ sample area, indicating the absence of rotational domains.

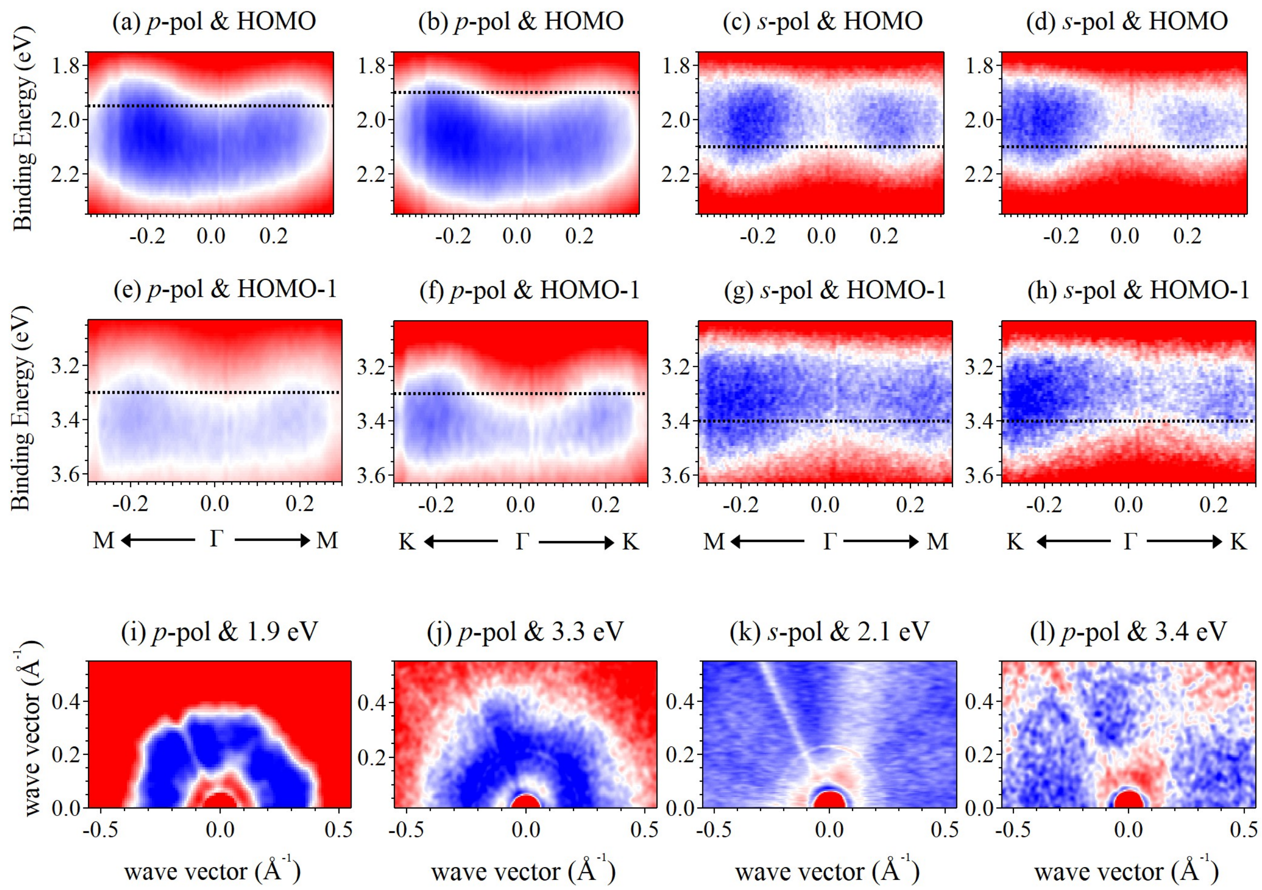

Figure S1: (a-h) ARPES spectra of C_{60}/Bi_4Te_3 taken around the energy of the HOMO and HOMO-1 bands for p-polarized and s-polarized light along ΓK and ΓM directions. (i-l) ARPES equi-energy maps of the HOMO and and HOMO-1 bands for p-polarized and s-polarized light.

C_{60} ($>$ 95.5% HPLC) was evaporated from a Knudsen cell at 675 K with an evaporation rate of 0.5 Åmin^{-1} as calibrated by a quartz microbalance (QMB). During evaporation, the substrate temperature was held at 400 K and post-annealed at 420 K for 30 min. ARPES measurements in Figure 1 were performed at the laboratory using an MBS A1 analyzer and a He gas discharge lamp. Data shown in Figure 2 and the SI were recorded on an identically prepared sample with a slightly different carrier concentration. ARPES spectra in Figures 2 and the SI were measured using synchrotron radiation with a photon energy of 21 eV at the BaDElPh beamline (Elettra).[4] Raman and PL spectra shown in Figure 2 were measured on the identical sample used for ARPES inside an optical cryostat. During Raman and PL

measurements, the vacuum inside the optical cryostat was better than 10^{-6} mbar and a green laser (532 nm) with a laser power of 5 mW was used.

Details of the calculation

For the C_{60} molecule adsorbed onto the Bi_4Te_3 surface, we considered a (3x3) in plane TI unit cell that is large enough to avoid molecule-molecule interaction of the neighboring unit cells. The spin-polarized calculations were performed by using DFT [5] and the projector augmented plane wave method[6] as implemented in the VASP code. [7,8] A cutoff energy of 500 eV was used for the plane wave expansion of the Kohn-Sham wave functions. [9] The structural relaxations were performed using vdW-DF2[10] functional with a revised Becke (B86b) exchange[11,12] to properly account for the nonlocal correlation effects like van der Waals interactions. [13] The analysis of the electronic structure was done by using the PBE exchange-correlation energy functional. [14] For the C_{60} molecule adsorbed onto the Bi_4Te_3 surface we considered a (3x3) in plane TI unit cell that is large enough to avoid molecule-molecule interaction of the neighboring unit cells.

Interaction energies of C_{60}/Bi_4Te_3

Unfaulted Bi_4Te_3 surface

By calculating the difference between the total energies of the combined system (C_{60}/Bi_4Te_3) and the sum of the isolated parts (i.e. C_{60} and Bi_4Te_3) as relaxed onto the substrate, we can evaluate the interaction energy of the C_{60} adsorbed onto the Bi_4Te_3 surface. Performing a similar analysis of the contributions to the total interaction energy as reported previously[15], we find a negative interaction energy due to non-local correlation effects of -1.240 eV while the DFT contribution is positive and amounts to $+0.524$ eV. These values suggest that the C_{60} molecule is physisorbed onto the surface with a total interaction energy of -0.716 eV.

Triple Te vacancy

In the case of a Te triple vacancy on the surface of Bi_4Te_3, the C_{60} molecule sits 1.10 Å above the surface with respect to the geometric position of a Te surface atom corresponding to an ideal terminated Bi_4Te_3 substrate. Note that, the shortest C-Te distance is 3.37 Å while the shortest C-Bi distance is 3.57 Å. That is, by adsorbing the C_{60} onto a triple Te vacancy, the molecule is lowered with 1.99 Å. As a consequence, the adsorption of C_{60} onto a triple vacancy leads to an interaction energy of -1.229 eV which also implies that the molecule-surface interaction becomes much stronger. Additionally, we find a negative interaction energy due to non-local correlation effects of -2.128 eV while the DFT contribution is positive and amounts to $+0.899$ eV. These values suggest that the C_{60} molecule is physisorbed onto the a triple vacancy of the TI surface with a total interaction energy of -1.229 eV.

Hole-doping to TI surface

The calculated Fermi surface area of due to the surface state measured from pristine TI sample and C_{60}/TI samples are 0.0776 $Å^{-2}$ and 0.0657 $Å^{-2}$, respectively. The area of two dimensional hexagonal unit cell of TI surface is ~51.47 $Å^{-2}$ taking the lattice constant of TI unit cell equal to 4.451 Å. The Brillouin zone area for the hexagonal unit cell is 1.68088 $Å^{-2}$. Note that, the Fermi surface of Bi_4Te_3 is not spin-degenerate and hence we do not have the usual factor 2 for spin-degeneracy below. The carrier concentration due to surface state for a pristine sample surface is

$$\begin{aligned}
\text{Carrier concentration } (\sigma) &= (\text{FS Area /BZ Area}) \times (\text{No. of unit cell / cm}^2) \\
\text{Carrier concentration from TI} &= (0.0776/1.6809) \times (10^{16} \ / \ 51.47\ ) \\
\sigma_{TI} &= 8.98 \times 10^{12} \text{ electrons/cm}^2
\end{aligned}$$

Similarly, the estimated carrier concentration form the surface state for C_{60}/Bi_4Te_3 is

$$\text{Carrier concentration from TI} = (0.0657/1.6809) \times (10^{16} \ / \ 51.47\ )$$

$$\sigma_{C_{60}/TI} = 7.59 \times 10^{12} \text{ electrons/cm}^2$$

Hence, the electron transfer from TI surface state to deposited C_{60} film or hole doped to TI surface state is 1.39 $\times 10^{12}$ electrons/cm^{-2}. The number of C_{60} unit cells/cm^2:

$$C_{60} \text{ unit cell/cm}^2 = 10^{16} \ / \ (3\sqrt{3}a_{C_{60}}^2 \ / \ 2)$$

$$= (10^{16} \times 2) / \ (3\sqrt{3}9.79)$$

$$= 4.016 \times 10^{13}$$

$$\text{Charge transfer per } C_{60} = (1.39 \times 10^{12}) \ / \ (4.016 \times 10^{13})$$

$$= 0.0346 \ e^{-}/C_{60}$$

The C_{60} lattice constant was estimated from the (4×4) superstructure of C_{60} on (9×9) Bi_4Te_3 grid observed from LEED spectra.

Evaluation of the density of triple Te vacancies

We assume that a fraction x of the C_{60} is on a triple Te vacancy and a fraction $(1 - x)$ is on an unfaulted (Te terminated) Bi_4Te_3 surface. The experimentally (ARPES) determined average electron transfer from Bi_4Te_3 to C_{60} is equal to 0.035 electrons per C_{60}. The DFT calculated electron transfer from the unfaulted Te terminated Bi_4Te_3 surface to C_{60} is equal to -0.042 electrons per C_{60}. The DFT calculated electron transfer from the triple vacancy of Te on the Bi_4Te_3 surface to C_{60} is equal to 0.2 electrons per C_{60}. That is, we have the equation $0.035 = 0.2x - 0.042x$ which yields $x = 0.22$. Thus, if we assume no other defects on the surface (such as anti-site defects in which a Te surface atom is replaced by Bi), we have one out of five C_{60} on top of a triple Te vacancy.

Dependence of charge transfer on the C_{60}-Bi_4Te_3 distance

Let us now look at the charge transfer from Bi_4Te_3 to C_{60} as a function of the height of C_{60} measured with respect to the surface of Bi_4Te_3. We describe the height of C_{60} by z, its position relative to equilibrium. For the equilibrium position, i.e. $z = 0$, we find that 0.208 electrons are transferred from Bi_4Te_3 to one C_{60}. The dependence of the charge transfer between C_{60} and Bi_4Te_3 as a function of z is plotted in Figure S2 for several z values between $z = -0.1$ (i.e. the C_{60} molecule is closer to the Bi_4Te_3 surface than in equilibrium) and $z = 0.3$ Å. From Figure S2 we observe that, the charge transfer decreases with increasing value of z.

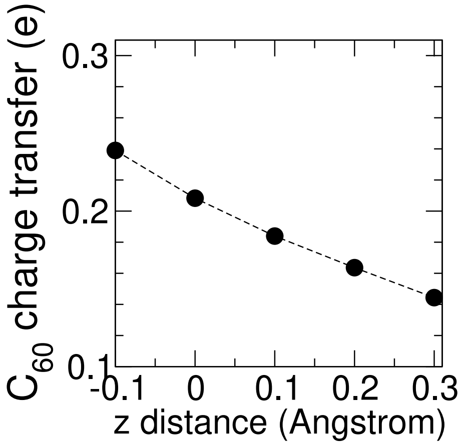

Figure S2: Calculated electron transfer from C_{60} to Bi_4Te_3 as a function of z movement of the C_{60} molecule out of the equilibrium position. The z direction corresponds to the direction perpendicular to the Bi_4Te_3 surface. The value of $z = 0$ on the horizontal axis corresponds to the energetic minimum of the C_{60} molecule.

References

(1) Dresselhaus, M.; Dresselhaus, G.; Eklund, P. Science of Fullerenes and Carbon Nanotubes. **1996**,

(2) Nabok, D.; Tas, M.; Kusaka, S.; Durgun, E.; Friedrich, C.; Bihlmayer, G.; Blügel, S.;

Hirahara, T.; Aguilera, I. Bulk and surface electronic structure of Bi_4Te_3 from *GW* calculations and photoemission experiments. Phys. Rev. Mater. **2022**, 6, 034204.

(3) Yamana, K.; Kihara, K.; Matsumoto, T. Bismuth tellurides: BiTe and Bi4Te3. Acta Crystallogr. B **1979**, 35, 147–149.

(4) Petaccia, L.; Vilmercati, P.; Gorovikov, S.; Barnaba, M.; Bianco, A.; Cocco, D.; Masciovecchio, C.; Goldoni, A. BaD ElPh: A 4m normal-incidence monochromator beamline at Elettra. Nuclear Instruments and Methods in Physics Research Section A: Accelerators, Spectrometers, Detectors and Associated Equipment **2009**, 606, 780–784.

(5) Hohenberg, P.; Kohn, W. Inhomogeneous electron gas. Phys. Rev. **1964**, 136, B864.

(6) Blöchl, P. E. Projector augmented-wave method. Phys. Rev. B **1994**, 50, 17953.

(7) Kresse, G.; Hafner, J. Ab initio molecular dynamics for liquid metals. Phys. Rev. B **1993**, 47, 558.

(8) Kresse, G.; Furthmüller, J. Ab initio molecular dynamics for liquid metals. Phys. Rev. B **1996**, 54, 11169.

(9) Kohn, W.; Sham, L. J. Self-consistent equations including exchange and correlation effects. Phys. Rev. **1965**, 140, A1133.

(10) Lee, K.; Murray, E. D.; Kong, L.; Lundqvist, B. I.; Langreth, D. C. Higher accuracy van der Waals density functional. Phys. Rev. B **2010**, 82, 081101.

(11) Becke, A. On the large-gradient behavior of the density functional exchange energy. J. Chem. Phys. **1986**, 85, 7184.

(12) Hamada, I. Higher-accuracy van der Waals density functional. Phys. Rev. B **2014**, 89, 121103.

(13) Huttmann, F.; Martinez Galera, A. J.; Caciuc, V.; Atodiresei, N.; Schumacher, S.; Standop, S.; Hamada, I.; Wehling, T. O.; Blügel, S.; Michely, T. Tuning the van der Waals Interaction of Graphene with Molecules via Doping. Phys. Rev. Lett. **2015**, 115, 236101.

(14) Perdew, J. P.; Burke, K.; Ernzerhof, M. Generalized gradient approximation made simple. Phys. Rev. Lett. **1996**, 77, 3865.

(15) Busse, C.; Lazić, P.; Djemour, R.; Coraux, J.; Gerber, T.; Atodiresei, N.; Caciuc, V.; Brako, R.; N'Diaye, A. T.; Blügel, S.; Zegenhagen, J.; Michely, T. Graphene on Ir(111): Physisorption with Chemical Modulation. Phys. Rev. Lett. **2011**, 107, 036101.
